# Posterior membranous tracheal injury during mckeown oesophagectomy. A case report with literature review

**DOI:** 10.3389/fonc.2025.1560437

**Published:** 2025-05-09

**Authors:** Theeran Kaur Gill, Guo Hou Loo, Guhan Muthkumaran, Nik Ritza Kosai

**Affiliations:** ^1^ Department of Surgery, Hospital Chancellor Tuanku Muhriz, National University of Malaysia, Kuala Lumpur, Malaysia; ^2^ Faculty of Medicine, The National University of Malaysia, Kuala Lumpur, Malaysia

**Keywords:** tracheobronchial injury, minimally invasive oesophagectomy, airway injury repair, cardillo classification, postoperative care in tracheal injury, thoracic surgery complications

## Abstract

Minimally invasive techniques such as thoracoscopic or robotic surgical approaches for oesophageal pathologies have been gaining traction as the preferred method of surgical technique. McKeown’s minimally invasive oesophagectomy has been shown to reduce hospitalisation, with reduced cardiopulmonary morbidities. However, it is not without complications, and an iatrogenic tracheobronchial injury (TBI) could occur intraoperatively during anatomical plane dissection. We report a case of iatrogenic posterior membranous tracheal injury during the thoracic dissection of a McKeown’s oesophagectomy, detected intraoperatively and patient recovered without any complications. The diagnosis of TBI involves a multicentric approach. Confirmation of the diagnosis and classification of TBI based on clinical signs, radiological studies, and endoscopy procedures such as bronchoscopy are necessary to tailor the best possible management for the patient. In cases where a full-thickness airway defect exceeds 2 cm and is detected intraoperatively, immediate primary repair is advised to optimize outcomes. TBI pose significant clinical challenges, particularly in cases of iatrogenic injury during procedures such as minimally invasive oesophagectomy. While the overall incidence of TBI remains low, awareness of risk factors and vigilant monitoring during procedures is paramount. While TBI remains rare, its management shares principles with oncological oesophageal surgery, making this case pertinent to surgical oncology practice. The evolving landscape of diagnostic techniques, including bronchoscopy and advanced imaging modalities, facilitates prompt and accurate identification of injuries, enabling timely intervention.

## Introduction

McKeown’s minimally invasive oesophagectomy (MIE) is comprised of a three–stage approach. It begins with the thoracoscopic mobilisation of the oesophagus followed by gastric conduit formation and cervical oesophagogastric anastomosis. Although often performed for oesophageal malignancies, MIE is increasingly utilised for benign conditions like achalasia cardia, especially with megaoesophagus, where surgical challenges parallel those encountered in cancer resections. To date, several studies that compared the outcomes of open oesophagectomy (OE) and MIE have been published, whereby most of them reported that MIE reduced surgical access-related trauma, which resulted in shorter hospitalisation, lower rates of respiratory complications and wound infections (1–3).

Given the anatomy of the structures in the neck and thorax with underlying pathologies, an iatrogenic tracheal injury could occur intraoperatively during anatomical plane dissection. Goldstein first documented an iatrogenic tracheal injury in 1949, which occurred during a jugular venepuncture when a needle inadvertently punctured the trachea (4). The incidence of tracheal injuries is relatively low around 1 – 10% while most of it is associated with the posterior membranous portion of the trachea (5).

This report highlights the intraoperative management of TBI during MIE for achalasia, drawing parallels with oncological oesophagectomy to inform surgical practice. This case has been reported in line with the SCARE criteria (6).

## Case presentation

**Figure 1:**
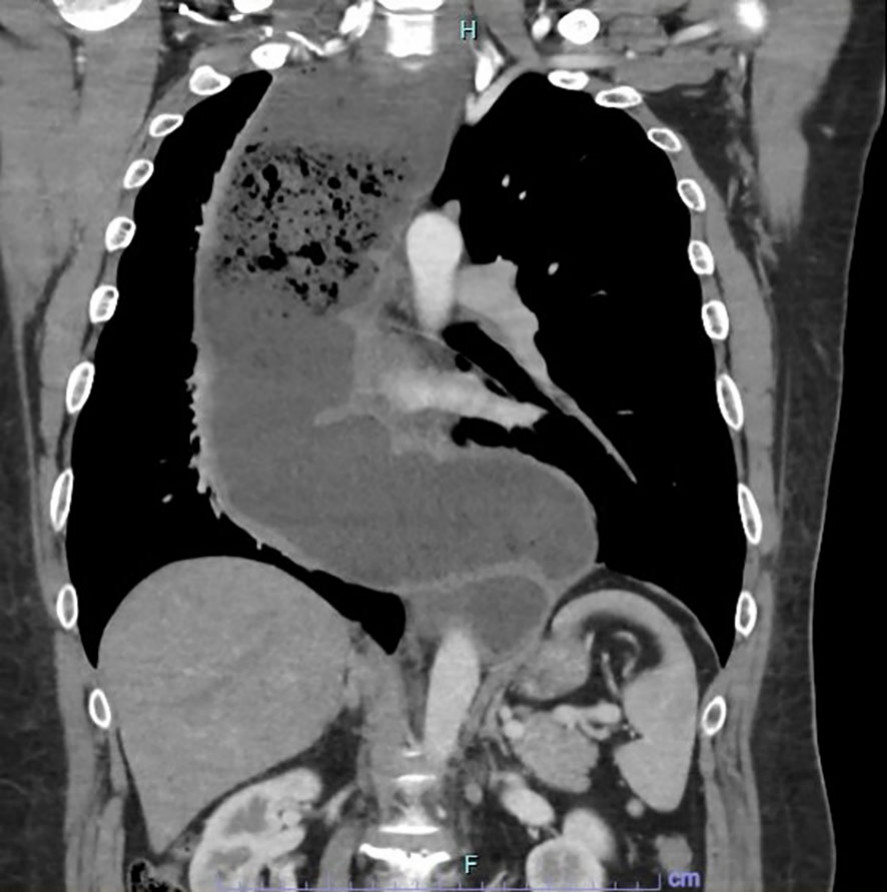
CECT thorax and abdomen in coronal view showing the tortuous and dilated oesophagus throughout its length measuring approximately 8.0cm in its maximum diameter with food particles and air-fluid levels noted within suggestive of end-stage achalasia with megaoesophagus.

A 42-year-old gentleman was planned to undergo MIE for a megaesophagus secondary to Type 1 achalasia cardia at our centre. The preoperative oesophagogastroduodenoscopy showed a grossly dilated oesophagus with retained fluid/food particles and puckering of the oesophago-gastric junction. The contrast-enhanced computer tomography of the thorax and abdomen revealed a tortuous, dilated oesophagus throughout its length measuring approximately 8.0cm in its maximum diameter with food particles and air fluid levels noted within suggestive of Type 1 achalasia with megaoesophagus (**Figure 1**). Prehabilitation was carried out through enteral tube feeding, chest physiotherapy with intensive spirometry, multidisciplinary approach consultations and family counselling for perioperative patient care.

**Figure 2:**
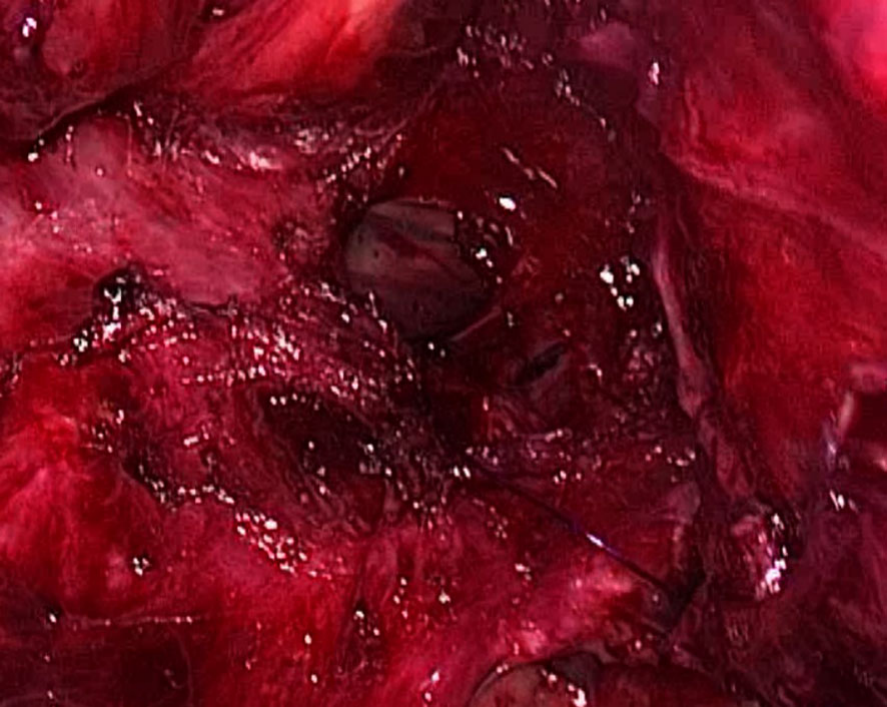
Thoracoscopic intraoperative image showing a 3cm defect at the posterior membranous part of the trachea.

**Figure 3:**
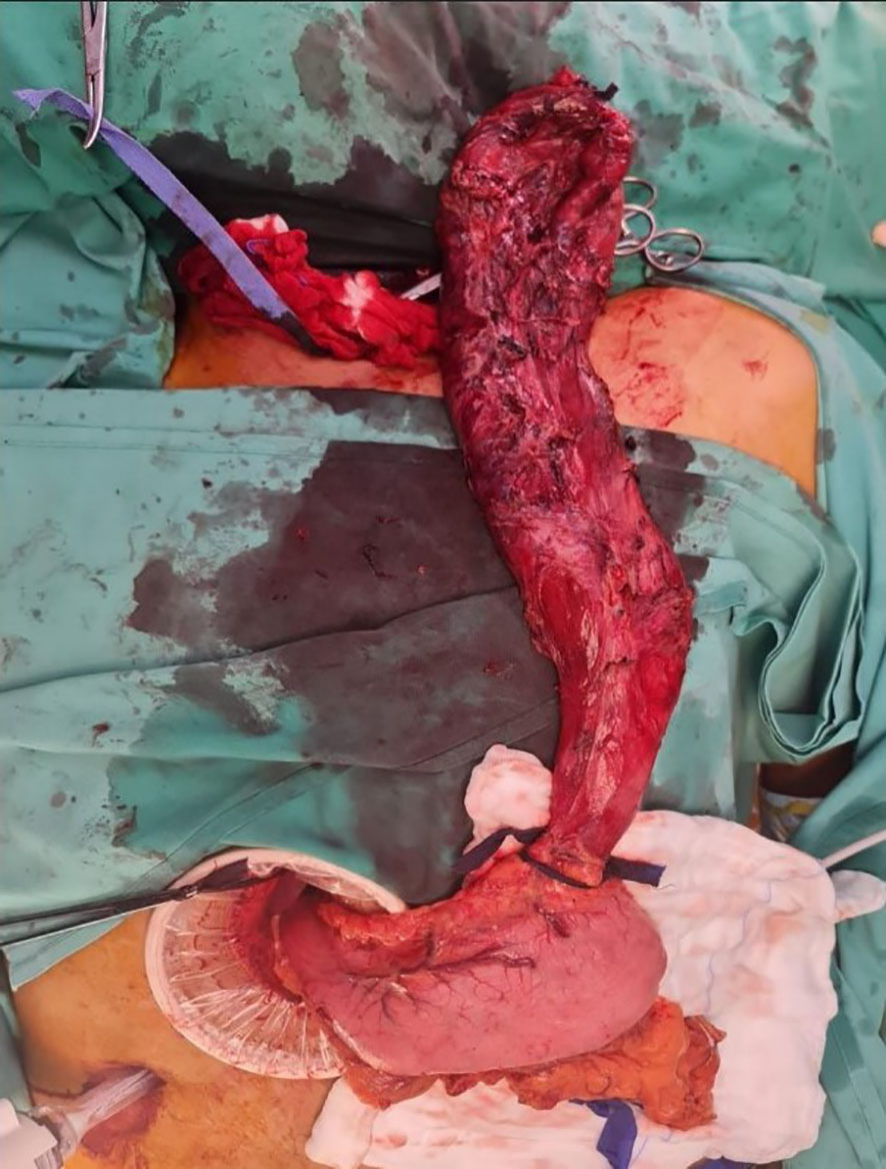
On table image showing the oesophagectomy specimen (still attached to the stomach) being delivered via a transabdominal approach.

The three-stage MIE was carried out three weeks post-prehabilitation with a double-lumen endotracheal tube. The abdominal dissection was carried out without difficulties. On thoracoscopic view, the oesophagus appeared tortuous and grossly dilated. There was the presence of dense adhesions with difficulty in dissecting the anatomical planes between the oesophagus, pleura and membranous portion of the trachea. An iatrogenic injury to the posterior membranous part of the trachea was observed during dissection measuring approximately 3 cm in length (**Figure 2**). Primary closure of the defect by interrupted sutures with polydioxanone 4/0 and local transposition of a pleural patch. Post repair, a jacuzzi test was performed by instillation of 0.9% saline around the area of interest and there was no leak demonstrable. The right lung was inflated with an intercostal drain *in situ*. Subsequently, the cervical anastomosis was completed with meticulous dissection. Subsequently, the cervical anastomosis was completed with meticulous dissection. However, because of the bulky specimen, it was delivered via a transabdominal approach (**Figure 3**). After the procedure, a bronchoscopy was performed by the attending anaesthesiologist to directly visualise the approximated edges of the membranous trachea. Post-surgery, the patient was admitted to the intensive care unit. Lung protective strategy with low tidal volumes and minimal airway pressures (positive end-expiratory pressure of 5-6cmH20) to prevent suture dehiscence were deployed. The patient remained intubated for 48 hours. A repeat bronchoscopy was performed before extubation, which showed an intact repair with no areas of leakage or suture disruption. A fluoroscopy was performed on day three, which showed good contrast flow, no leakage or hold-up of contrast within the conduit. In-hospital, daily assessment included airway patency, voice quality, and respiratory function. The patient was discharged home on day 10 post-op with no respiratory complications. Outpatient reviews at 2 weeks, 1 month, 3 months, and 6 months included clinical examination and flexible nasal endoscopy. At 6 months, the patient was asymptomatic with no hoarseness or airway stenosis.

## Discussion

Tracheobronchial injuries (TBI) are rare but potentially high-impact events with significant morbidity and mortality. They are defined as those that occur between the cricoid cartilage and the right and left mainstem tracheal bifurcation. Common aetiologies include blunt or penetrating trauma and iatrogenic injury that might occur during surgery, stressful endotracheal intubation, tracheostomy or rigid bronchoscopy. Most iatrogenic tracheal injuries are longitudinal tears to the membranous part of the trachea. In other incidences, these tears may also occur on the side that pulls the membranous part of the trachea away from the cartilage (7).

Statistically significant recent data related to thoracoscopic iatrogenic tracheal injury is not available (8). Intraoperative TBI occurs most frequently during oesophageal surgery for middle and proximal oesophageal tumours, with the incidence of TBI occurring during transhiatal oesophagectomy ranging from 0.4 to 0.6% (9, 10). The incidence of TBI during routine endotracheal intubation is low, approximately 0.005% for single-lumen intubation and 0.05% for double-lumen intubation (11). However, the incidence can be as high as 15% following difficult intubation, while the incidence of TBI following tracheostomy placement is approximately 0.2% (12, 13).

Additional contributors to iatrogenic tracheal injury include advanced patient age, long-term corticosteroid use, and prior treatment with chemoradiotherapy, locally advanced upper and mid-oesophageal tumours, oesophageal squamous cell carcinoma, presence of peri-tumoural inflammation, performing an extended mediastinal lymphadenectomy and female sex (5, 8, 14). Procedure-related causes, such as direct surgical injury in open or laparoscopic surgeries and double-lumen ETT insertions, are also implicated in airway injuries (8, 15).

The initial clinical signs of TBI are expanding mediastinal and subcutaneous emphysema, pneumomediastinum, pneumothorax, ventilatory resistance or high air leakage along the endotracheal cuff and bloody secretions from the tube. If the patient was extubated, persistent coughing, shortness of breath and haemoptysis are highly suspicious of TBI (16, 17). Pneumothorax, due to the splitting of the mediastinal pleura, occurs in 17–70%, and the most specific characteristic is the inability to re-expand the lung after thoracostomy. There may be the presence of a rare clinical manifestation called Hamman’s sign or a mediastinal “crunch” which is a crackling sound, synchronous with heartbeats, may be heard over the precordium produced by the heart beating against air-filled tissues (18). Delayed clinical signs usually present within four weeks post-injury are haemoptysis and pneumonitis complicating an obstructed airway. Rarely, do patients present with a healed airway injury years later, typically with dyspnoea or with the diagnosis of newly diagnosed bronchial asthma (19).

The diagnosis of TBI involves a multicentric approach. Confirmation of the diagnosis and classification of TBI based on clinical signs, radiological studies, and endoscopy procedures such as bronchoscopy are necessary to tailor the best possible management for the patient. A plain chest X-ray may reveal signs of tracheal rupture, such as ‘lung fall syndrome,’ characterized by downward displacement of the lung due to loss of bronchial tethering. This syndrome prompts the loss of bronchial suspension so that the injured lung decreases to the cardio-diaphragmatic angle, which is different from the compression of normal lung tissue to the hilum of the lung for pneumothorax (20).

However, CT of the neck and thorax is preferable in comparison to chest radiography. In cases of tracheal injury, CT of the neck and thorax may reveal pneumomediastinum, subcutaneous emphysema, pneumothorax or tracheal tear (21). The characteristic findings of tracheal rupture on CT are gas dispersion around the broken ends, bronchial lumen stenosis or blockage, and bronchial displacement or angular deformity (22). Signs such as increased attenuation of the mediastinal fat pad and mediastinal fluid collection can be appreciated on CT thorax to diagnose post-tracheal injury complications such as acute mediastinitis (23).

For patients who are unable to tolerate bronchoscopy, we can implement the multi-planar reformation of two-dimensional or three-dimensional reconstruction with simulation bronchoscopy technology which can show the airway disease in the conventional axial CT imaging. As for cases with severe chest trauma whose CT is negative but highly suspected bronchial injury, the technique of three-dimensional reconstruction of MDCT can be used to reconstruct the bronchial tree for the diagnosis of complete bronchus rupture (20).

Bronchoscopy remains the gold standard for the diagnosis of tracheal injury. It not only helps in identifying the exact location and size of the injury but may also help in the treatment of the injury and can be performed intraoperatively in the occurrence of an iatrogenic tracheal injury during surgery (24). In patients who are endotracheally intubated, the endotracheal cuff may obscure the injured trachea and hinder its assessment. In such patients, the diagnosis of tracheal injury may be delayed. In such circumstances or cases with high suspicion of tracheal injury, the endotracheal cuff should be deflated during bronchoscopic evaluation to assess the extent and severity of the injury (25).

Cardillo et al. developed a morphological grading system to stratify tracheal injuries by the extent of wall involvement to standardize the options for treatment. A Level I tracheal injury involves mucosal or submucosal injury without mediastinal emphysema and oesophageal injury. Level II is when the lesion extends into the muscular wall with subcutaneous or mediastinal emphysema but without oesophageal injury or mediastinitis. Level IIIa involves complete laceration with oesophageal or mediastinal soft-tissue herniation without oesophageal injury or mediastinitis. Lastly, level IIIb includes any laceration with oesophageal injury or mediastinitis (26).

Our case corresponds to Level IIIa, given the full-thickness tracheal tear with herniation risk but no mediastinitis. Given our intraoperative finding, primary repair with pleural buttress was appropriate. Early intraoperative bronchoscopy confirmed repair adequacy, while postoperative bronchoscopies ensured ongoing integrity. Postoperative ventilation strategy was critical; low tidal volumes reduced airway pressures across the repair. Serial bronchoscopy and imaging provided reassurance before extubation and feeding commencement. Importantly, this case underscores that even in benign oesophageal pathology, risks and surgical strategies mirror those in oncologic surgery. The principles of airway injury management are applicable across both spectrums.

Early recognition of clinical signs and symptoms can help risk-stratify patients and guide treatment. The main aspects for treatment decisions are based on the length of the injury, the amount of air leakage with pneumothorax, the possibility of bypassing the injury without expanding it with a blocked cuff, and the amount of mediastinal contents protrusion into the lumen. In addition to that, the current condition and prognosis of the patient is important for decision-making (25).

The management of tracheal injuries can be categorized into conservative and surgical strategies. Conservative treatment is generally considered for patients with Level I or II injuries who remain clinically stable. These patients may either breathe independently or need only minimal ventilatory support and typically present with tracheal lacerations measuring 2 cm or less. Additional favourable criteria include absence of oesophageal involvement, limited mediastinal air, non-progressive subcutaneous emphysema or pneumomediastinum, and unsuitability for surgery due to other medical considerations (25, 27).

In contrast, surgical intervention is advised for those with Level IIIa or IIIb injuries, particularly if there is increasing subcutaneous emphysema, pneumothorax, pneumomediastinum, or persistent air leakage, as well as failure of lung re-expansion despite chest drainage. Other surgical indications include herniation of the oesophageal wall into the tracheal lumen, ineffective ventilation due to a distal tracheal tear, or intraoperative recognition of a tracheal injury, as was the scenario in our case (25, 28).

The approach to conservative treatment includes patient observation with antitussive medications. These medications are given to prevent persistent coughing as it may lead to further extension of the tracheal tear and disrupt local healing. In these instances, broad spectrum antibiotics according to the local hospital protocol can be initiated although evidence is limited (29). In patients requiring prolonged mechanical ventilation with uncertain outcomes, placing a ventilation tube under bronchoscopic guidance to bypass the injury site can be beneficial. It is essential that the tube’s cuff is positioned beyond the injury zone, in an area of intact tracheal tissue. When this is not feasible, a double-lumen endotracheal tube may be employed temporarily for approximately 3 to 4 days. Within this timeframe, smaller tracheal defects often become sealed by a fibrin layer that aids in initial wound closure (16). For Level II and IIIa injuries, endoscopic application of fibrin glue can serve as a supportive treatment, while endobronchial stenting is increasingly recognized as a viable non-surgical option for iatrogenic tears measuring between 2 to 4 cm (26).

In patients who are not suitable candidates for surgical repair, tracheal stenting offers a less invasive alternative with potential to expedite healing, despite being associated with a relatively higher rate of perioperative complications. These stents are effective and technically straightforward to deploy, typically allowing early tissue recovery. Removal is generally advised at six weeks, as granulation tissue formation tends to increase after the three-month mark. Nonetheless, complications such as stent migration, mucus accumulation, unpleasant breath, and tissue overgrowth have been observed. Reports suggest that the use of fully covered or silicone-based stents may reduce the frequency of such events (30). Another conservative option is tracheostomy, which helps by lowering airway pressure and minimizing air leakage at the injury site, thereby promoting spontaneous closure of the tear (31).

Surgical repair of tracheal injuries is guided by several fundamental principles: ensuring sufficient surgical exposure, maintaining an appropriate airway segment for effective debridement and reconstruction, preserving the lateral vascular supply to the trachea to prevent ischemia and impaired anastomotic healing, and retaining the option to perform a tracheostomy when necessary. Additionally, reinforcement of the repair, often via tissue buttressing, plays a key role in preventing dehiscence. The surgical access may range from a traditional right thoracotomy to a transcervical approach, or more recently, a hybrid transcervical-transtracheal technique employing a T-shaped tracheal incision to enhance visibility and access (25, 32).

For lesions affecting the upper or middle third of the trachea, a low cervical collar incision (cervicotomy) is frequently adequate. This method allows thorough evaluation of adjacent structures such as the oesophagus and major blood vessels, while offering the benefits of a less invasive approach (33, 34). In contrast, injuries to the mid-trachea may necessitate splitting the manubrium via a T-shaped incision to facilitate evaluation and management of potential vascular involvement. Injuries involving the left main bronchus are optimally approached through a left-sided thoracotomy, whereas distal tracheal tears extending toward or beyond the carina, or involving the main bronchi, generally require a right thoracotomy, occasionally in combination with a cervicotomy for optimal exposure.

Extensive access procedures such as thoracosternotomy or bilateral “clamshell” incisions, which are typically reserved for penetrating trauma, are seldom required in the context of iatrogenic injuries (25). Nonetheless, when thoracotomy is needed for distal airway repair, it may increase the risk of postoperative complications, including mortality, as observed in certain studies reporting a 2.2% mortality rate following surgically treated airway injuries (35, 36).

Definitive surgical repair is strongly advised when a tracheal injury identified intraoperatively involves the full thickness of the tracheal wall or exceeds 2 cm in length, criteria that were met in the case we present (35). When such an injury occurs during minimally invasive oesophagectomy, it may necessitate conversion to an open approach, which carries a heightened risk of postoperative pulmonary complications. Nevertheless, recent evidence suggests that minimally invasive modalities, including thoracoscopic and robotic techniques, offer comparable long-term oncologic outcomes to open surgery in the management of oesophageal malignancies. These approaches are also linked to fewer cardiopulmonary adverse events and improvements in postoperative quality of life (37).

## Conclusion

Tracheobronchial injuries (TBI) pose significant clinical challenges, particularly in cases of iatrogenic injury during procedures such as minimally invasive oesophagectomy. While the overall incidence of TBI remains low, awareness of risk factors and vigilant monitoring during procedures is paramount. Despite the non-oncological nature of the case, the surgical complexities and management strategies offer valuable insights for oesophageal surgeons, particularly those dealing with malignancies. Surgical management, guided by the extent and nature of the injury, has seen advancements, with minimally invasive approaches offering promising outcomes and reduced postoperative complications.

## Data Availability

The original contributions presented in the study are included in the article/supplementary material. Further inquiries can be directed to the corresponding author.
